# Characteristics, survival and neurological outcome in out-of-hospital cardiac arrest in young adults in Sweden: A nationwide study

**DOI:** 10.1016/j.resplu.2023.100503

**Published:** 2023-11-10

**Authors:** Linnea Gustafsson, Araz Rawshani, Truls Råmunddal, Björn Redfors, Petur Petursson, Oskar Angerås, Geir Hirlekar, Elmir Omerovic, Christian Dworeck, Sebastian Völz, Johan Herlitz, Clara Hjalmarsson, Lina Dahlén Holmqvist, Anna Myredal

**Affiliations:** a University of Gothenburg, Institute of Medicine, Department of Molecular and Clinical Medicine, Sweden; b Sahlgrenska University Hospital, Department of Emergency Medicine, Gothenburg, Sweden; c Sahlgrenska University Hospital, Department of Cardiology, Gothenburg, Sweden; d The Swedish Cardiopulmonary Resuscitation Registry, Centre of Registries, Västra Götaland, Gothenburg, Sweden

**Keywords:** Cardiac arrest, Cardiovascular disease, OHCA, Survival

## Abstract

**Aim:**

The aim of this study was to present a comprehensive overview of out-of-hospital cardiac arrests (OHCA) in young adults.

**Methods:**

The data set analyzed included all cases of OHCA from 1990 to 2020 in the age-range 16–49 years in the Swedish Registry of Cardiopulmonary Resuscitation (SRCR). OHCA between 2010 and 2020 were analyzed in more detail. Clinical characteristics, survival, neurological outcomes, and long-time trends in survival were studied. Logistic regression was used to study 30-days survival, neurological outcomes and Utstein determinants of survival.

**Results:**

Trends were assessed in 11,180 cases. The annual increase in 30-days survival during 1990–2020 was 5.9% with no decline in neurological function among survivors. Odds ratio (OR) for heart disease as the cause was 0.55 (95% CI 0.44 to 0.67) in 2017–2020 compared to 1990–1993. Corresponding ORs for overdoses and suicide attempts were 1.61 (95% CI 1.23–2.13) and 2.06 (95% CI 1.48–2.94), respectively. Exercise related OHCA was noted in roughly 5%. OR for bystander CPR in 2017–2020 vs 1990–1993 was 3.11 (95% CI 2.57 to 3.78); in 2020 88 % received bystander CPR. EMS response time increased from 6 to 10 minutes.

**Conclusion:**

Survival has increased 6% annually, resulting in a three-fold increase over 30 years, with stable neurological outcome. EMS response time increased with 66% but the majority now receive bystander CPR. Cardiac arrest due to overdoses and suicide attempts are increasing.

## Highlights

• The annual relative increase in 30-days survival is approximately 6%, which has resulted in a 3-fold increase in survival over the past three decades.
• 88% of all cases in this study now receive bystander CPR, which is the highest figure reported to date.
• Almost 20% of the OHCA in the youngest age group are caused by attempted suicide and intoxication/overdose is seen in 30% of individuals between 16–34 years. These etiologies are increasing while heart disease is rapidly diminishing.

## Introduction

Out-of-hospital cardiac arrest (OHCA) is a leading cause of mortality worldwide and a majority of OHCAs occur in the older population.^1^ Most OHCAs occurs due to cardiac causes, with an increasing prevalence for coronary heart disease (CAD) with age.^2^, ^3^ Young adults represent a minority of OHCAs and the cause of cardiac arrest in this population is more heterogeneous, although CAD seems to be the strongest risk factor.^4^, ^5^, ^6^, ^7^, ^8^, ^9^ Previous research on OHCA in young adults has frequently emphasized on athletes, and demonstrated that cardiovascular conditions are the most common causes of cardiac arrest during exercise. A wide range of etiologies underly these events, including hypertrophic obstructive cardiomyopathy, channelopathies, congenital heart disease, and coronary artery disease.^10^, ^11^ However, other common causes of OHCA in younger individuals include suicide attempts and drug overdoses.^12^, ^13^

The aim of this study was to provide a comprehensive view of cardiac arrest in young adults, aged 16 to 49 years, which includes the age-range where CAD is a common cause. We assessed both trends in survival and characteristics, as well as predictors of survival.

## Methods

Ethical approval was obtained by the Swedish Ethical Review Authority (2019–01094).

The funders had no role in the study.

### Study population

The Swedish Registry of Cardiopulmonary Resuscitation (SRCR) is a nationwide healthcare quality registry launched in 1990. All emergency medical services (EMS) in Sweden participate in the registry and report all cases of out of hospital cardiac arrest (OHCA) where resuscitation has been initiated, using the Utstein style of reporting.^14^ In Sweden, there is a two-tiered EMS system that responds to all medical emergencies. The first tier provides basic level of life support, and units in the second tier provide advanced life support. In cases of suspected cardiac arrest, two ambulances are dispatched, and dispatchers instruct the caller to perform cardiopulmonary resuscitation.^15^ The registry and variable definitions have been described previously.^15^, ^16^

The data set used to study trends in characteristics and outcomes included all cases of OHCA registered in the SRCR in individuals aged 16 to 49 years from 1990 to 2020, 11,180 cases in total. The coverage and completeness of data reported to the register have improved over time and the level of ascertainment has been > 90% since 2010. To provide further details on clinical characteristics, management and outcomes, a separate dataset was used. This included cases aged 16 to 49 years who were enrolled during 2010 through 2020 (6358 cases among 11,180 who were registered during the period), which were linked to the Prescribed Drug Register, Inpatient Register, Outpatient Register, Cause of Death Register and the Socioeconomic Data Register. The linkage is seamless through every Swedish citizen’s unique 12-digit personal identification number which provides individual level information regarding coexisting conditions, medications, socioeconomic status, and causes of death, see Table 1.

**Table 1:** Baseline characteristics in 6358 patients with out of hospital cardiac arrest during 2010–2020 in relation to age-group.

	**16 to 24 years**	**25 to 34 years**	**35 to 44 years**	**45 to 49 years**
N	1029	1704	1962	1663
Women	309 (30.0)	461 (27.1)	569 (29.1)	500 (30.1)
Age - mean (SD)	20.93 (2.42)	29.52 (2.85)	39.88 (2.91)	47.21 (1.40)
**Cause of cardiac arrest**				
Heart disease	93 (10.1)	172 (11.3)	457 (26.7)	602 (40.8)
Overdose or intoxication	249 (26.9)	494 (32.5)	298 (17.4)	117 (7.9)
Trauma or accident	103 (11.1)	125 (8.2)	94 (5.5)	61 (4.1)
Pulmonary disease	12 (1.3)	22 (1.4)	38 (2.2)	60 (4.1)
Suffocation	27 (2.9)	45 (3.0)	65 (3.8)	50 (3.4)
Suicide	178 (19.2)	210 (13.8)	185 (10.8)	103 (7.0)
Drowning	42 (4.5)	29 (1.9)	29 (1.7)	25 (1.7)
Other	221 (23.9)	422 (27.8)	544 (31.8)	457 (31.0)
**Born abroad**	121 (11.9)	238 (14.2)	399 (20.9)	327 (20.1)
**Coexisting conditions**				
Affective disorders	222 (21.6)	551 (32.3)	504 (25.7)	316 (19.0)
Hypertension	13 (1.3)	44 (2.6)	179 (9.1)	285 (17.1)
Obesity	30 (2.9)	96 (5.6)	150 (7.6)	125 (7.5)
Type 2 diabetes	3 (0.3)	24 (1.4)	84 (4.3)	166 (10.0)
Heart failure	19 (1.8)	44 (2.6)	51 (2.6)	111 (6.7)
Renal failure	18 (1.7)	47 (2.8)	79 (4.0)	78 (4.7)
Dyslipidemia	15 (1.5)	68 (4.0)	69 (3.5)	61 (3.7)
Type 1 diabetes	12 (1.2)	32 (1.9)	69 (3.5)	99 (6.0)
Chronic ischemic heart disease	4 (0.4)	10 (0.6)	42 (2.1)	88 (5.3)
Cardiomyopathies	27 (2.6)	27 (1.6)	38 (1.9)	50 (3.0)
Acute myocardial infarction	2 (0.2)	10 (0.6)	36 (1.8)	85 (5.1)
**Medications prescribed**				
Beta blockers	35 (3.4)	84 (4.9)	130 (6.6)	215 (12.9)
ACE inhibitor or ARB	17 (1.7)	54 (3.2)	119 (6.1)	246 (14.8)
Anticoagulant or antiplatelet agent	13 (1.3)	31 (1.8)	95 (4.8)	172 (10.3)
Antidiabetic drugs	12 (1.2)	31 (1.8)	88 (4.5)	137 (8.2)
Diuretics	13 (1.3)	35 (2.1)	75 (3.8)	135 (8.1)
Lipid lowering drugs	4 (0.4)	11 (0.6)	58 (3.0)	141 (8.5)
Calcium channel blockers	3 (0.3)	13 (0.8)	52 (2.7)	97 (5.8)
**Location of cardiac arrest**				
Home	584 (57.0)	1060 (62.5)	1214 (62.2)	1041 (63.0)
Public place	290 (28.3)	398 (23.5)	454 (23.3)	376 (22.7)
Other places	151 (14.7)	238 (14.0)	284 (14.5)	236 (14.3)
**Witnessed cardiac arrest**	394 (40.5)	684 (41.7)	960 (51.1)	899 (56.1)
**Exercise within 60 min before cardiac arrest**	17 (4.1)	21 (2.8)	39 (4.9)	33 (5.5)
**Prehospital interventions**				
Bystander CPR	632 (63.5)	1048 (64.0)	1130 (60.3)	974 (60.2)
Telephone CPR	178 (58.9)	353 (64.8)	367 (63.9)	332 (70.6)
AED used by bystander	8 (22.2)	12 (16.9)	25 (33.8)	21 (38.9)
Mechanical compressions	366 (36.8)	669 (41.1)	782 (41.5)	596 (37.4)
Defibrillated any	212 (21.4)	313 (19.4)	575 (30.5)	605 (37.8)
Adrenaline administered	756 (74.3)	1243 (74.6)	1441 (74.2)	1287 (78.3)
Amiodarone administered	48 (4.8)	99 (6.0)	184 (9.6)	213 (13.1)
**Critical time intervals (**median IQR)				
Time from arrest to EMS arrival	12.0 [8.0, 19.0]	13.0 [9.0, 19.0]	13.0 [8.0, 9.0]	13.0 [9.0, 20.0]
Time from EMS dispatch to arrival	10.0 [6.0, 15.0]	10.0 [6.0, 15.0]	10.0 [6.0, 6.0]	10.00 [7.0, 17.0]
**Initial rhythm**				
VF/pVT	123 (14.3)	176 (12.5)	383 (22.6)	421 (28.5)
PEA	90 (10.5)	135 (9.6)	194 (11.5)	164 (11.1)
Asystole	648 (75.3)	1100 (78.0)	1115 (65.9)	893 (60.4)
**OUTCOMES**				
ROSC, any	331 (34.0)	510 (31.6)	647 (34.8)	535 (33.4)
Survival at 30 days	207 (20.1)	305 (17.9)	373 (19.0)	292 (17.6)

VF = ventricular fibrillation, pVT = pulseless ventricular tachycardia, PEA = pulseless electrical activity, ROSC = return of spontaneous circulation

The Inpatient Register and Outpatient Register include the primary and all secondary discharge diagnoses in inpatient and outpatient care throughout Sweden. The Inpatient Register includes hospitalizations since 1987 and has been validated.^17^ The Outpatient Register contains all outpatient visits since 2002. Both registries employed the 10th revision of the International Classification of Disease (ICD) during the study period. Prescribed drugs have been recorded in the Swedish Prescribed Drug Register since 2005. All prescriptions in ATC (Anatomical Therapeutic Chemical) classes A, B and C (with three characters detail) since 1st Jan 2008 were retrieved. Socioeconomic data was retrieved from the LISA database (longitudinal integrated database for health insurance and labor market studies).

### Exposures

All individuals were categorized into the following age groups: 16 to 24 years, 25 to 34 years, 35 to 44 years, and 45 to 49 years. Individuals aged 0 to 15 years are in Sweden handled in pediatric care and were therefore not included in this study.

### Definition of coexisting conditions

For all diagnoses, the first date of diagnosis (in primary or secondary position) was retrieved from the Inpatient Register or the Outpatient Register.

### Outcomes

The primary outcome measure was 30-day survival. The secondary outcome measure was neurological function measured using cerebral performance category (CPC) scale. The CPC score was assessed at discharge and ranged from 1 to 5 (1, no sequelae; 2, mild sequelae; 3, severe sequelae; 4, vegetative state; 5, brain dead).

### Descriptive statistics

Baseline characteristics were described using means and medians with appropriate measures of dispersion.

### Long-term trends 1990–2020

Logistic regression, adjusted for age and sex, was used to compute probabilities and odds ratios (OR) for presenting with shockable rhythm, etiology and 30-day survival. OR were calculated by comparing the first four calendar years (1990-1993, reference period) with the final four calendar years (2017–2020). We compared, by means of odds ratios, the first and final time periods across all trends. Critical time intervals and rates of bystander CPR were not adjusted, in order to provide the actual figures. Multivariable logistic regression was used to study the association between key characteristics (e.g. initial rhythm) and 30-day survival in the population included during 2010–2020.

## Results

### Long-term trends 1990–2020

#### Trends in survival, etiology, initial presentation and critical time intervals

**Fig. 1:**
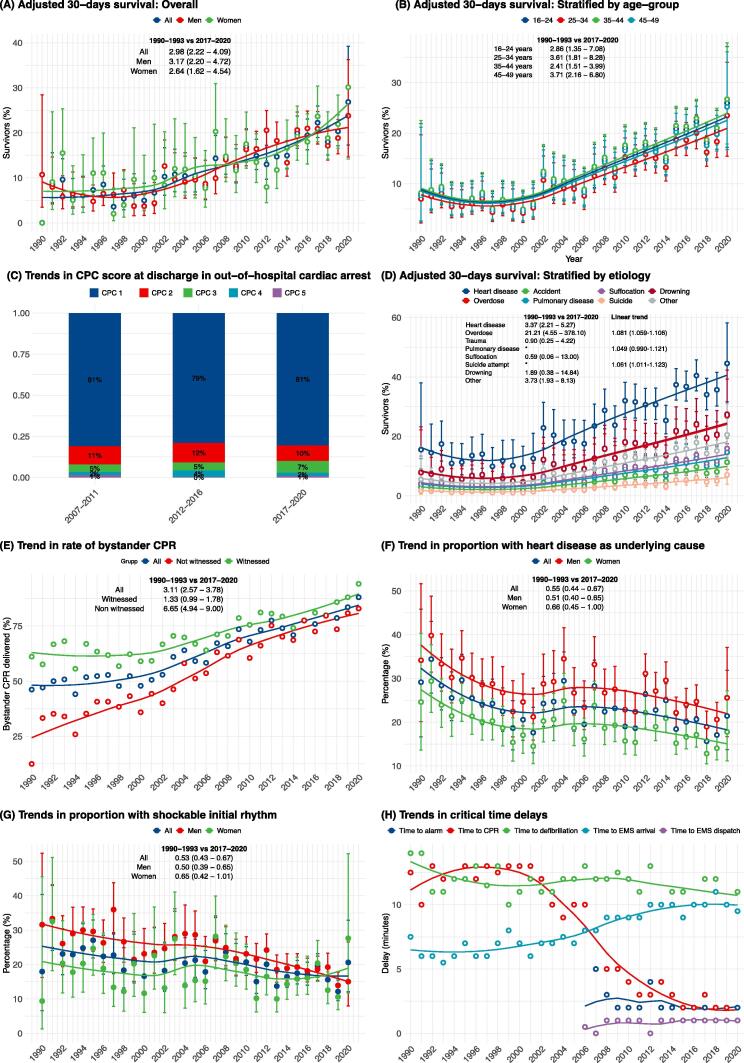
Trends in 30-day survival, clinical characteristics, and critical time intervals during 1990 to 2020.

Fig. 1A-H shows trends from 1990 to 2020. During 1990 to 2000, there were no significant changes in 30-day survival. Survival increased linearly between year 2000 and 2020. The annual increase in 30-day survival during the entire study period was 5.9% (95% CI 1.6% to 7.1%) overall, 6.3% (95% CI 5.3% to 7.3%) in men and 4.9% (95% CI 3.5% to 6.4%) in women. Men did not display a significant improvement in survival in the final decade, whereas women did (11.4% per year). OR for 30-day survival in 2017–2020 vs 1990–1993 was 2.98 (95% CI 2.22 to 4.09) overall, 3.17 (95% CI 2.20–4.72) in men and 2.64 (95% CI 1.62–4.54) in women (Fig. 1A).

With regards to age-groups, survival increased similarly across all groups (Fig. 1B). CPC score 1 (no sequelae) or 2 (mild sequelae) was 92% among survivors during 2007–2011, as compared with 91% in 2017–2020, with no significant difference (Fig. 1C).

Regarding etiologies, increased survival was seen for individuals with OHCA due to heart disease (6.5% [5.3%-7.8%]), overdose (8.1% [5.9%-10.6%]), suicide attempts (6.1% [1.1%–12.3%]), and other causes (6.7% [5.0%–8.5%]). OR for 30-day survival in cases caused by heart disease in 2017–2020 vs 1990–1993 was 3.37 (95% CI 2.21 to 5.27) (Figure D).

Bystander CPR was performed in roughly 50% of cases during 1990 to 2000 (Fig. 1E). In 2020, bystander CPR was performed in 88%, 94% in women and 81% in men. The annual increase was 4.8% (4.3%-5.3%), 1.3% (0.5%-2.0%) in witnessed cases and 8.3% (7.5%-9.1%) in non-witnessed cases. Overall, OR for bystander CPR in 2017–2020 vs 1990–1993 was 3.11 (95% CI 2.57 to 3.78).

OR for overdose as the underlying cause in 2017–2020 vs 1990–1993 was 1.61 (95% CI 1.23–2.13). OR for suicide attempt as the underlying cause in 2017–2020 vs 1990–1993 was 2.06 (95% CI 1.48–2.94). The probability of heart disease as the underlying cause was halved during the study period (OR for 2017–2020 vs 1990–1993 was 0.55 [95% CI 0.44 to 0.67]). In 1990–1993 approximately 32% of the cases were caused by heart disease, compared with roughly 18% in the final four years (Fig. 1F). In parallel, the probability of shockable initial rhythm was halved during the study period (Fig. 1G).

Time from collapse to CPR (no-flow time) was approximately 11 minutes during the first years and decreased to around 2 minutes in the final four years. This improvement took place during 2000 to 2010, whereas no-flow remained relatively unchanged before and after that period. Time to defibrillation declined from 14 to 11 minutes whereas time to EMS arrival increased from 6 minutes to 10 minutes (Fig. 1H).

### Characteristics and survival during 2010–2020

#### Characteristics

In total 6,358 cases were recorded in the SRCR during 2010–2020. There were 1,029 individuals aged 16–24 years, 1,704 aged 25 to 34 years, 1,962 aged 35 to 44 years and 1,663 aged 45 to 49 years. The proportion of women was approximately 30% in all age groups (Table 1).

#### Underlying cause of OHCA

Heart disease as the underlying cause increased with age, varying between 10.1% to 40.8%. Suicide attempts, drowning, trauma, and accidents were more common underlying causes among the youngest OHCA individuals. Overdose or intoxication varied from 7.9% (age-group 45 to 49 years) to 32.5% (age-group 25–34 years).

#### Coexisting chronic conditions

A previous diagnosis of affective disorders was most frequent in individuals aged 25–34 years: 32.3% (Table 1). Hypertension had been diagnosed in 17.1% of individuals aged 45–49 years, as compared with 1.3% of those aged 16–24 years. Obesity was previously diagnosed in 7.5% of those aged 45–49 years, compared with 2.9% in the youngest age group. In individuals aged 45–49 years, heart failure had been previously diagnosed in 6.7%, renal failure in 4.7%%, acute myocardial infarction in 5.1% and any form of ischemic heart disease in 5.3%.

#### Exercise related cardiac arrests

Exercise related OHCA was noted in 4.1%, 2.8%, 4.9% and 5.5% in cases aged 16 to 24 years, 25 to 34 years, 35 to 44 years, and 45 to 49 years, respectively (Table 1).

#### Bystanders, initial rhythm, and circulation

Bystander CPR was overall performed in 60.2% to 64.0%, somewhat more frequently in the younger age-groups. Cardiac arrests were more frequently witnessed in the older age-groups. Ventricular fibrillation (VF) or pulseless ventricular tachycardia (pVT) were found in 14.3%, 12.5%, 22.6% and 28.5% in cases aged 16 to 24 years, 25 to 34 years, 35 to 44 years, and 45 to 49 years, respectively. Rates of pulseless electrical activity (PEA) were comparable across all groups. Time from collapse to EMS arrival was 13 minutes (median) in all groups, except the youngest group in which the median delay was 12 minutes (Table 1).

#### Outcome, ROSC and 30-day survival in relation to time to CPR

Any return of spontaneous circulation (ROSC) occurred in 34.0%, 31.6%, 34.8% and 33.4% in individuals aged 16 to 24 years, 25 to 34 years, 35 to 44 years, and 45 to 49 years, respectively (Table 1). When time from collapse to CPR was <3 minutes, 64.4% of cases 16–24 years had ROSC, compared with 47.7% in those aged 45–49 years. Rates of ROSC decreased linearly with increasing no-flow time.

#### 30-day survival

**Fig. 2:**
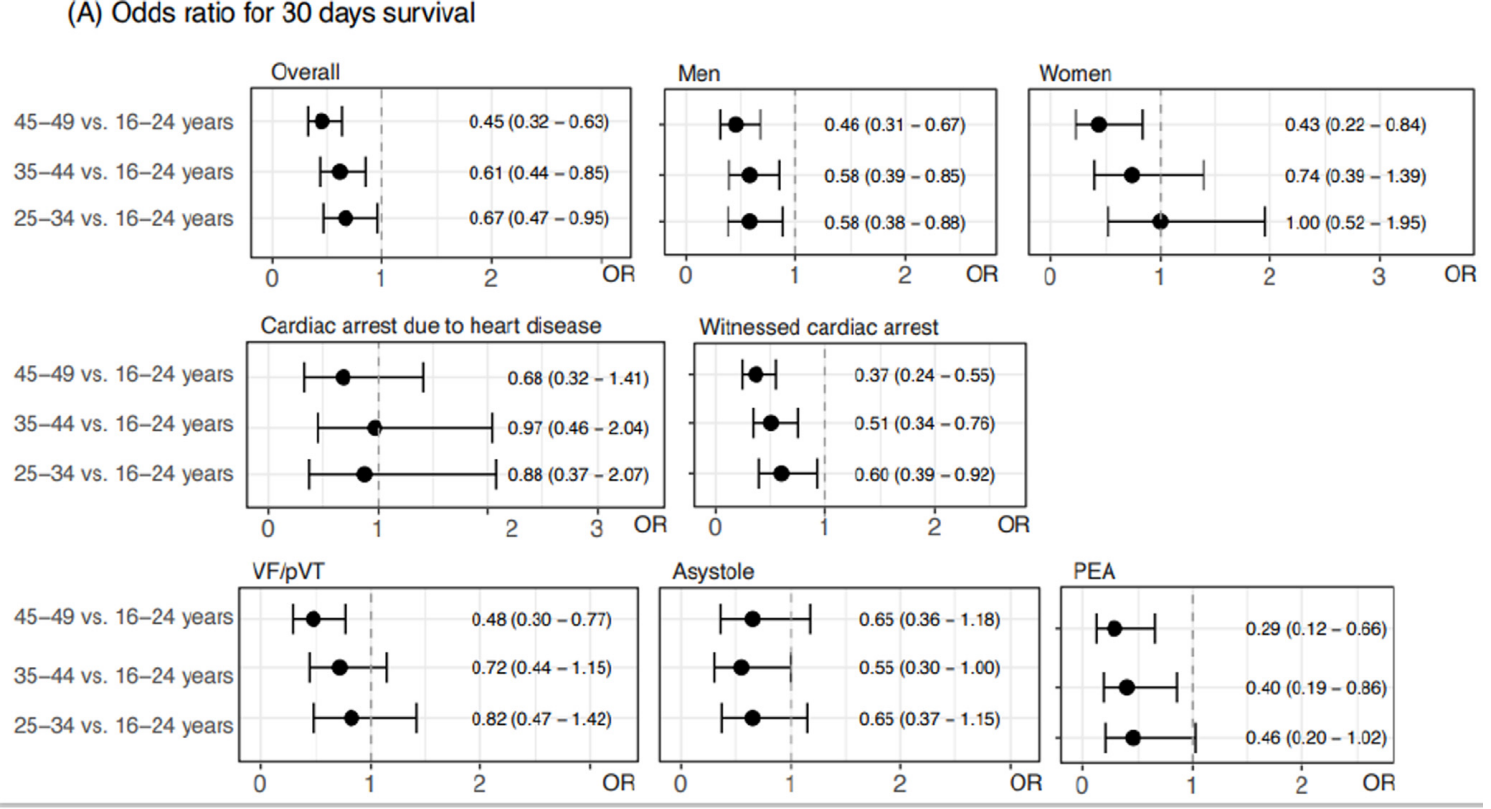
Odds ratios for 30-day survival in relation to age-group, adjusted for sex, location, no-flow time, and initial rhythm.

After adjusting for sex, location, no-flow time and initial rhythm, odds ratios for 30-day survival were 0.45 (0.32–0.63) for individuals aged 45–49 years, 0.61 (0.44–0.85) for individuals aged 35–44 years, 0.67 (0.47–0.95) for individuals aged 25–34 years, all compared with those aged 16–24 years. These associations were also evident in subgroups related to sex, witnessed status and initial rhythm (Fig. 2).

#### Neurological outcome

**Fig. 3:**
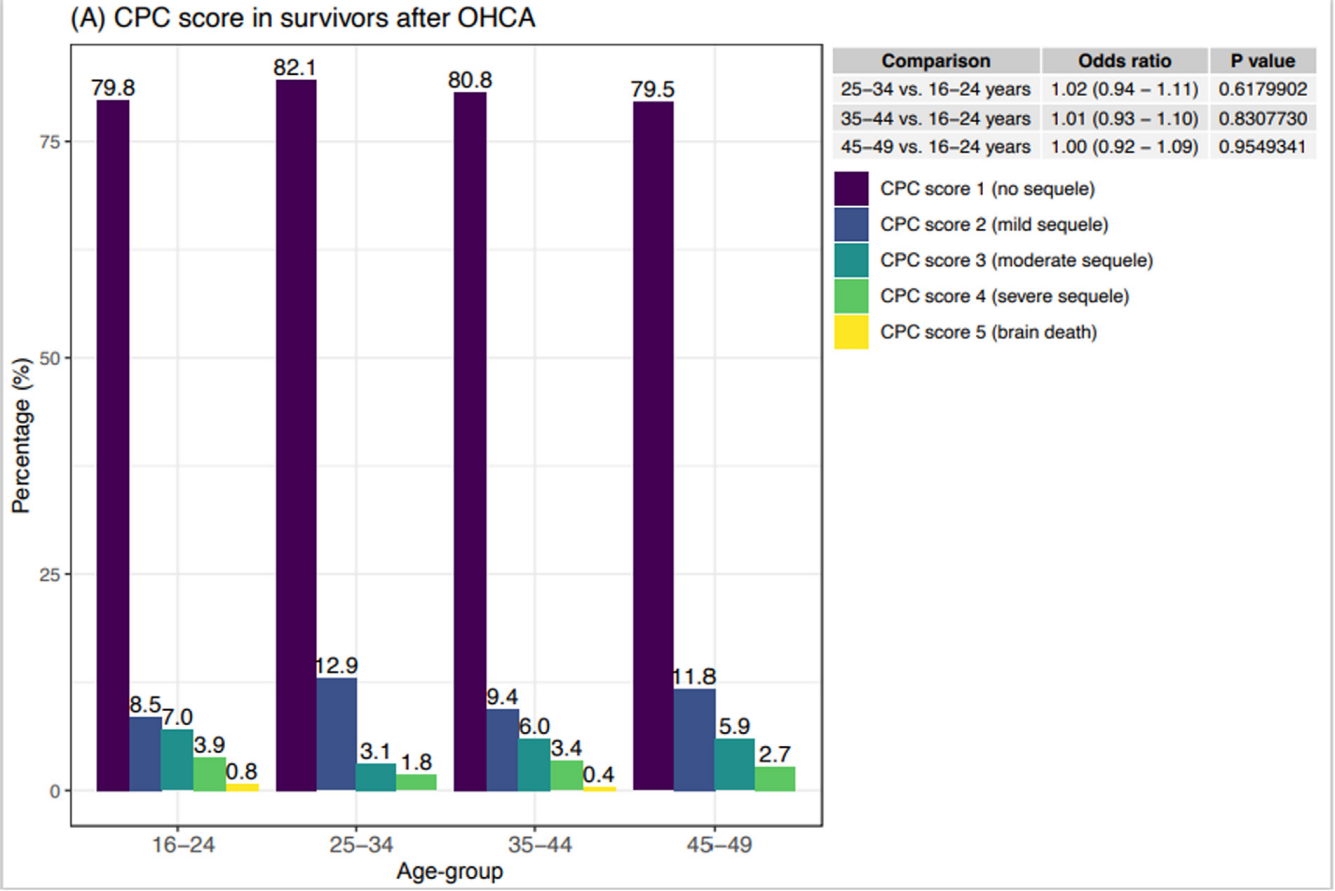
Cerebral performance category (CPC) score among survivors of out-of-hospital cardiac arrest 2010–2020. Odds ratios (right upper panel) for exhibiting CPC 1 at discharge, adjusted for sex, location, no-flow time, and initial rhythm.

There was no significant difference between the age groups in term of neurological outcomes (Fig. 3).

#### Predictors of 30-day survival

**Fig. 4:**
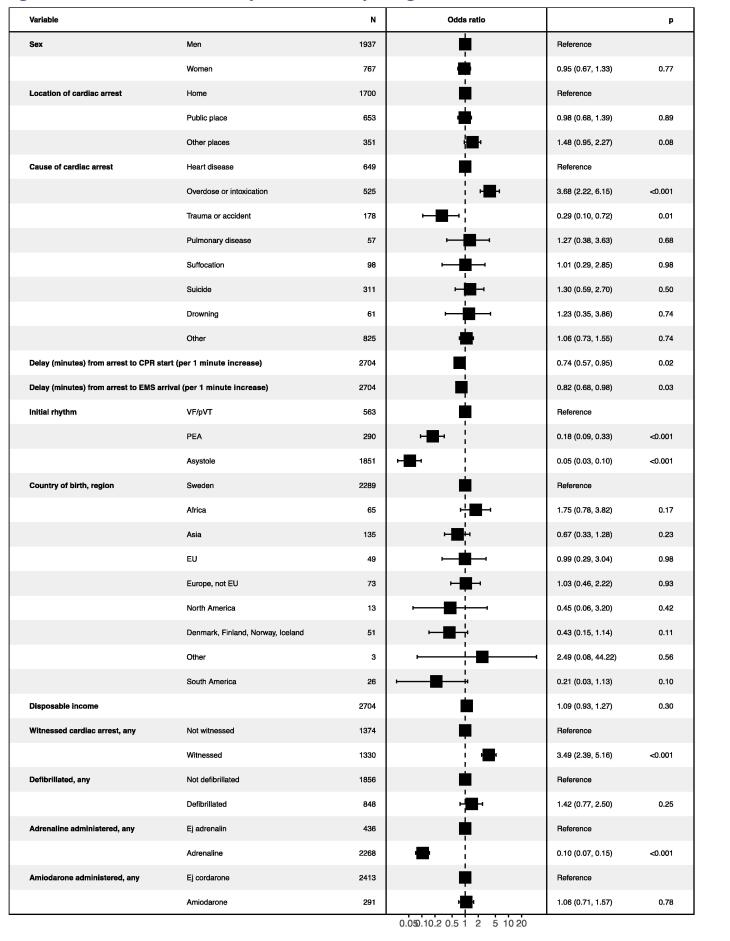
Odds ratios for 30-day survival among young adults experiencing an out-of-hospital cardiac arrest (OHCA) 2010–2020.

Fig. 4 shows predictors of 30-day survival. Sex had no significant association after accounting for the predictors presented in Fig. 4. Trauma was associated with poorer outcome, whereas overdose/intoxication was associated with better outcomes, as compared with heart disease. As compared with presenting with VF/pVT, odds ratio for 30-day survival was 0.18 (0.09–0.33) for PEA and 0.05 (0.03–0.10) for asystole. Survival dropped by 26% per minute delay to CPR.

## Discussion

This study sheds light on 30 years of cardiac arrest in young adults in an entire nation. We show that survival is increasing rapidly, especially since year 2000. The annual relative increase in 30-day survival is approximately 6%, which has resulted in a 3-fold increase in survival over the past three decades. We also show that survival is increasing uniformly across the whole age-span from 16 to 49 years of age. While survival has increased dramatically, neurological function has remained good (CPC 1 or 2) in >90% of survivors over the entire study period, suggesting that more individuals are being saved without compromising neurological outcomes. These improvements noted in the young are more pronounced than the improvements noted for the general population.^18^

With regards to age-related differences in survival, we note substantial differences in 30-day survival within the young population studied here. Survival in patients aged 45–49 years was half that of survival in those 16–24 years. There was no difference in survival in relation to age when the initial rhythm was asystole, where the adjusted probability of survival was reduced by 95%.

Another finding in this study is that 88% of all cases now receive bystander CPR, which is the highest figure reported to date.^15^, ^18^, ^19^, ^20^ The high rate of bystander CPR is encouraging, and may be one explanation of the improvement of survival with remaining good neurological outcome.^15^, ^20^, ^21^ It is unclear whether present findings are applicable for other countries, where bystander CPR occurs less frequently, but can hopefully inspire to improve public education in CPR. The highest survival rates were noted for cases with heart disease as underlying cause, although the likelihood of having such etiology was halved during the study period.

The perception that young individuals who experience an OHCA often are athletes with structural or genetic heart disease is not backed by our data, which shows that sports-related cardiac arrests are uncommon across the age-span 16 to 49 years of age. The highest rate of exercise related OHCA found in our data was 1 in 20 events. While exercise may certainly trigger malignant arrhythmias^11^ it should in an unselected population be viewed as uncommon, which is in line with previous studies.^6^

In contrast to OHCA of cardiac etiologies, cardiac arrests due to overdoses and suicide attempts have increased sharply. Almost 20% of the OHCA in the youngest age group are caused by attempted suicide and intoxication/overdose is seen in about 30% of individuals between 16–34 years. This trend was described by Herlitz et al 2006 using data from SRCR from 1990-2004.^22^ Ågren et al showed that during the period 2000–2017, the mortality rate in young adults (20–34 years old) decreased by 42% in Western Europe, whereas in Sweden it remained constant. Furthermore, self-harm and substance use disorder as cause of death decreased in Western Europe during the same period, a decrease that was not seen in Sweden.^23^ Our data shows that up to one third of the individuals with OHCA have a history of affective disorders, which suggests a high prevalence of psychiatric disease compared to other studies^24^ but is consistent with the findings of increasing long-time trends in self-reported depressive and anxiety symptoms, especially among the young.^25^, ^26^ Both poor mental health and substance use in childhood and adolescence are known risk factors for cardiovascular disease.^23^, ^27^, ^28^, ^29^, ^30^ Another possibility could be that there is a correlation between psychiatric disease and the high rates of suicide attempts and intoxication, although there are no data in the present study to support this.

Our data also suggests that known cardiovascular disease, defined as any previous inpatient or outpatient diagnosis of cardiovascular conditions, was uncommon in the younger age range but more common in the higher age range, as expected. Obesity and diabetes type 2 were more frequent in the age group 45 to 49 years than in the population.^31^ The prevalence of previous acute myocardial infarction (MI) and ischemic heart disease were around 5% in the oldest age-group. This suggests that the majority of OHCAs caused by myocardial ischemia and infarction represent the first manifestations of coronary artery disease.^32^ The decline in prevalence and cardiovascular mortality in the studied population is perfectly aligned with the decline in the general Swedish population.^33^

The greatest improvements in survival occurred during the period 2000 to 2020. This overlaps with the introduction of dispatcher (telephone) assisted CPR (introduced in 1998), rapid dissemination of CPR training in Sweden, and the dissemination of public AEDs in society. This is corroborated by the rapid increase in bystander CPR as well as reductions in time to CPR and time to defibrillation. Ambulance response times doubled during the study period, which has previously been described,^34^ and is presumably an important contributor to the decline in shockable initial rhythm. As of 2020, survival appears to be increasing for all age-groups, all etiologies and both sexes. This is an encouraging finding that clearly suggests that current resuscitation protocols for OHCA in young adults in Sweden have been successful.

Strengths of this study are the nationwide coverage over time with the inclusion of >11,000 patients and the large amount of detailed data since 2010. Limitations include some uncertainty of compliance regarding reporting to the registries, especially in the earlier years, but also the observational design where residual confounding cannot be ruled out. Furthermore, this is a study conducted in Sweden and whether findings can be extrapolated to other countries is uncertain. However, this study bears a lot of important information that could help to improve the care of an important, not sufficiently studied group.

## Conclusions

30-day survival in OHCA in the young population is increasing 6% annually, resulting in a 3-fold increase in survival over 30 years, without compromising the neurological outcome among survivors. The improvement coincides with a dramatic increase in bystander CPR. This improvement is counteracted by a doubling of EMS response times during the same period. The high prevalence of pre-existing psychiatric diagnoses, together with the high share of suicide attempts and intoxications causing cardiac arrests in this young population are the most alarming findings in this study and indicates there is a major need for preventive interventions within this field.

## Sources of Funding

Swedish Research Council (2019–02019), Swedish state under the agreement between the Swedish government, and the county councils (ALFGBG-971482), The Wallenberg Centre for Molecular and Translational Medicine.

## Declaration of Generative AI and AI-assisted technologies in the writing process

All authors declare no conflicts.

## Author Contributions

LG, AM and AR conceived the study. LG and AR performed statistical analyses. LG drafted the first version of the manuscript. All authors contributed to all subsequent revisions of the manuscript, interpretation of data, and finalization of the manuscript including final approval of the submitted version.

## Declaration of competing interest

The authors declare that they have no known competing financial interests or personal relationships that could have appeared to influence the work reported in this paper.
