# Supplementary material for: Characteristics, survival and neurological outcome in out-of-hospital cardiac arrest in young adults in Sweden: A nationwide study
Source: Resusc Plus. 2023 Nov 10;16:100503. doi: 10.1016/j.resplu.2023.100503 (PMC10665903; doi:10.1016/j.resplu.2023.100503)
Supplement: Supplementary data 1 [file mmc1.docx]

**Supplementary Table 1. Missingness per variable, excluding variables with complete data**

| Variable | Percentage missing |
| --- | --- |
| Sex | 0.2 |
| Location of cardiac arrest | 0.5 |
| Bystander CPR, chest compressions performed | 0.5 |
| Bystander CPR, ventilations performed | 0.9 |
| Adrenaline | 1.4 |
| Intubation | 1.8 |
| Cause of cardiac arrest (old definition) | 11.5 |
| Time from EMS dispatch to EMS arrival | 13.9 |
| Initial rhythm | 14.4 |
| Work / profession category | 16.2 |
| Born in Sweden or abroad | 2.0 |
| Country of birth | 2.0 |
| Conscious on EMS arrival at scene | 2.6 |
| Amiodarone | 2.8 |
| Breathing on EMS arrival at scene | 2.8 |
| Clock time of OHCA (clock hour) | 22.7 |
| Time from CA to CPR start | 27.4 |
| Bystander CPR (new definition) | 3.6 |
| ROSC on arrival at hospital | 34.2 |
| Conscious on arrival at hospital | 35.5 |
| Time from CA to EMS arrival | 38.3 |
| Mechanical compressions | 4.0 |
| Defibrillated, any | 4.1 |
| Witnessed cardiac arrest, anyone | 4.1 |
| ROSC, any | 4.9 |
| Laryngeal mask placed | 40.3 |
| Hospitalized | 40.9 |
| Time from CA to EMS dispatch | 41.7 |
| Ambulance first on scene | 46.2 |
| Emergency service first on scene | 47.8 |
| Bystander connected AED | 48.4 |
| Police first on scene | 48.5 |
| Pulse on EMS arrival at scene | 5.4 |
| Bystander CPR (old definition) | 58.1 |
| Cardiac arrest during exercise/sports | 59.7 |
| Citizenship | 6.4 |
| Marital status | 6.4 |
| Telephone CPR | 70.3 |
| Discharged alive | 72.6 |
| Percutaneous Coronary Intervention performed | 73.7 |
| CABG performed | 73.7 |
| Defibrillations, number | 74.1 |
| ICD implantation during in-hospital stay | 74.1 |
| Time from CA to ROSC | 74.5 |
| Witnessed cardiac arrest, by ambulance | 75.2 |
| Time from CA to first defibrillation | 77.3 |
| Bystander education | 79.0 |
| Educational attainment | 8.1 |
| Location of CA - Public place - Details, public | 80.7 |
| Location of CA - Public place - Details, other places | 86.2 |
| CPC at discharge | 86.8 |
| Bystander used AED | 96.3 |
| Coronary angiography performed | 98.2 |
| ECMO | 98.2 |
